# A Review of Cell-Based Computational Modeling in Cancer Biology

**DOI:** 10.1200/CCI.18.00069

**Published:** 2019-02-04

**Authors:** John Metzcar, Yafei Wang, Randy Heiland, Paul Macklin

**Affiliations:** ^1^ Indiana University, Bloomington, IN

## Abstract

Cancer biology involves complex, dynamic interactions between cancer cells and their tissue microenvironments. Single-cell effects are critical drivers of clinical progression. Chemical and mechanical communication between tumor and stromal cells can co-opt normal physiologic processes to promote growth and invasion. Cancer cell heterogeneity increases cancer’s ability to test strategies to adapt to microenvironmental stresses. Hypoxia and treatment can select for cancer stem cells and drive invasion and resistance. Cell-based computational models (also known as discrete models, agent-based models, or individual-based models) simulate individual cells as they interact in virtual tissues, which allows us to explore how single-cell behaviors lead to the dynamics we observe and work to control in cancer systems. In this review, we introduce the broad range of techniques available for cell-based computational modeling. The approaches can range from highly detailed models of just a few cells and their morphologies to millions of simpler cells in three-dimensional tissues. Modeling individual cells allows us to directly translate biologic observations into simulation rules. In many cases, individual cell agents include molecular-scale models. Most models also simulate the transport of oxygen, drugs, and growth factors, which allow us to link cancer development to microenvironmental conditions. We illustrate these methods with examples drawn from cancer hypoxia, angiogenesis, invasion, stem cells, and immunosurveillance. An ecosystem of interoperable cell-based simulation tools is emerging at a time when cloud computing resources make software easier to access and supercomputing resources make large-scale simulation studies possible. As the field develops, we anticipate that high-throughput simulation studies will allow us to rapidly explore the space of biologic possibilities, prescreen new therapeutic strategies, and even re-engineer tumor and stromal cells to bring cancer systems under control.

## INTRODUCTION

Cancer is a complex systems problem that involves interactions between cancer cells and their tissue microenvironments.^1-3^ Therapeutic approaches that narrowly focus on cancer cells frequently lead to disappointing outcomes, including resistance, tissue invasion, and treatment failure. Such failures are partly due to the unexpected behaviors that emerge from the dynamical systems of cancer tissues. Therapies act as selective pressures, even while cancer cells use increased genetic variability to broadly sample survival strategies and adapt.^3,4^ Chronic hypoxia, another selective pressure, leads to metabolic changes, selection for cancer stem cells that resist treatment, invasion, and angiogenesis.^4-6^ Tumor cells communicate biochemically and biomechanically with stromal cells, which allows them to co-opt normal physiologic processes.^1-3,7,8^ Mathematical models can serve as "virtual laboratories" with fully controlled conditions where scientists and clinicians can investigate the emergent clinical behaviors that result from basic cell hypotheses and can evaluate new therapeutic strategies.^1,9^

This review surveys cell-based methods for simulating cancer. Also known as discrete models, agent-based models, or individual-based models, cell-based models simulate individual cell behaviors within tissue environments. These models have several advantages. Each cell agent can track a fully independent state with individual parameters that reflect heterogeneity in cancer. Modelers can directly implement cell rules that reflect observations of single-cell behavior and cell-cell interactions, which allow us to translate biologic hypotheses to mathematical rules quickly; run simulation experiments that explore the emergent behaviors of these hypotheses; and compare against new data to confirm, reject, or iteratively improve the underlying hypotheses.^1,9,10^

## A SURVEY OF CELL-BASED MODELING METHODS

**FIG 1.**
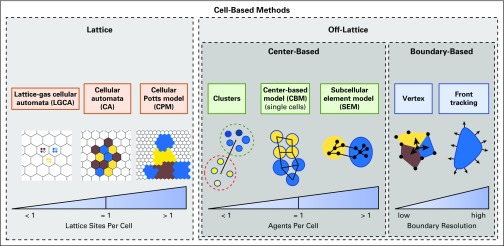
A schematic classification of cell-based modeling approaches.

Cell-based models represent individual cells with two main paradigms—lattice-based models that track cells along a rigid grid and off-lattice models that have no such restriction. Figure 1 classifies most cell-based modeling approaches. Table 1 lists major open source modeling packages.

**TABLE 1.**
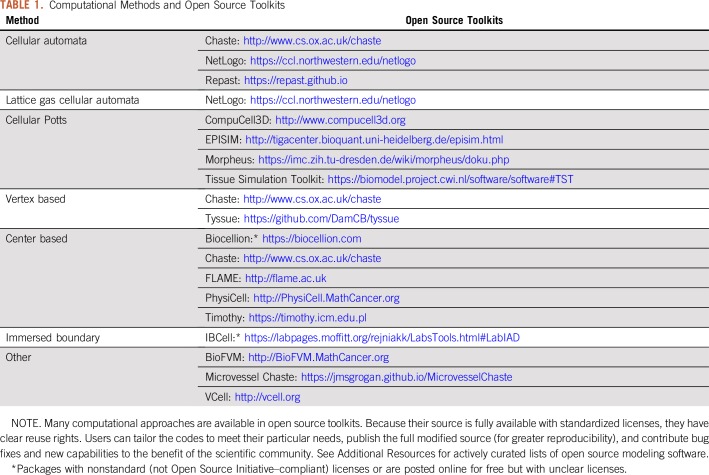
Computational Methods and Open Source Toolkits

### Lattice-Based Methods

Lattice-based models can use regular structured meshes (eg, Cartesian^11^ [two- or three-dimensional [2D/3D], dodecahedral [3D])^12^ or unstructured meshes.^13^ Structured meshes are simpler to implement, visualize, and combine with partial differential equation (PDE) solvers, but their structure can lead to grid biases.^13^ Unstructured meshes can avoid these issues^13^ but with greater complexity.

We can further categorize lattice-based methods by their spatial resolution. In cellular automaton (CA) models, each lattice site can hold a single cell.^14-17^ At each time step, each cell is updated with discrete lattice-based rules: remain, move to a neighboring lattice site, die (free a lattice site), or divide to place a daughter cell in a nearby site.^14-17^ These methods usually update the lattice sites in a random order to reduce grid artifacts.^14,15^

In lattice gas CA (LGCA) models, a single lattice site can contain multiple cells.^14,15,17,18^ LGCA models track the number of cells that move through channels between individual lattice sites rather than the motion of each individual cell. They can simulate very large numbers of cells efficiently over long periods while also connecting to statistical mechanics theory; this facilitates analysis and provides a bridge to continuum methods that model cell densities or populations instead of single cells.^17,18^

Some problems may require resolution of individual cell morphologies. Cellular Potts models (CPMs) use multiple lattice sites to represent each cell.^14,15,19^ At each time step, CPMs visit each pixel (2D) or voxel (3D), test a random swap with a neighboring pixel/voxel, and accept or reject the swap (probabilistically) on the basis of whether it would reduce a global energy. Although CPMs can model cell morphologies and mechanics that cannot be incorporated in CA models, they are much more computationally intensive. Also, the calibration of Monte Carlo steps to physical time can be challenging.^20^

### Off-Lattice Methods

We can divide off-lattice models into center-based models (CBMs) that focus on cell volumes (or masses) and models that focus on cell boundaries. We can further classify these approaches by level of morphologic detail.

#### CBMs.

CBMs track each cell’s center of mass or volume, typically by using a single software agent per cell.^13-15,21^ Some CBMs represent cells as points, whereas others explicitly model cell volumes. CBMs typically update the cells’ positions by explicitly formulating the adhesive, repulsive, locomotive, and drag-like forces exchanged between cell centers.^13-15,21^ Most CBMs approximate cells as spheres; however, some approximate cells as deformable ellipsoids to better represent their morphologies.^22,23^

CBMs can model cell morphology in greater detail by breaking cells into subcellular elements^24,25^: Each cell is represented by multiple center-based agents that interact with adhesive and repulsive forces. These models better approximate cell biomechanics but at increased computational cost. Conversely, cells can be organized into clusters or functional units (eg, breast glands or colon crypts) that are simulated as agents that interact by mechanical forces or other rule-based motions^26,27^; this allows modelers to incorporate heterogeneous details into individual clusters of cells but with greater computational efficiency than traditional CBMs.

#### Boundary-tracking models.

Vertex-based methods (eg, Fletcher et al^28^) model cells as polygons (2D) or polyhedra (3D) and compute the forces that act on their vertices; they are particularly useful for modeling confluent tissues.^29^ For greater spatial resolution, front-tracking methods, such as the immersed boundary method (IBM), solve PDEs for fluid flow inside and between cells and then advect boundary points along the cells’ membranes in this flow.^30^ Level set methods have been applied to implicitly track the movement of cell boundaries,^31^ and VCell (see Connecting to Molecular Effects) recently added front-tracking capabilities.^32,33^ These are among the most computationally intensive cell-based methods, but they are useful for coupling detailed cell mechanics to fluid and solid tissue mechanics.

### Connecting to Molecular Effects

Most cell-based models are hybrid discrete-continuum; they couple a discrete cell model to continuum models of the microenvironment.^1,14,15^ In general, these models use reaction-diffusion PDEs to simulate biotransport of oxygen, growth factors, and drugs. Ghaffarizadeh et al^34^ developed BioFVM to solve diffusive transport of tens to hundreds of chemical substrates in 3D tissues; it is the underlying PDE solver for PhysiCell (a center-based simulation framework).^21^ In this framework, modelers write rules to relate individual cell phenotypes to local chemical substrate conditions.^21^

Many discrete models include systems of ordinary differential equations (ODEs) to model molecular processes in individual cells.^35,36^ VCell can simulate reacting flows of many proteins within a single detailed cell,^32,33^ and many modeling packages (eg, Chaste,^37^ CompuCell3D,^38^ and EPISIM^39^) support systems biology markup language (SBML) to include systems of ODEs that simulate molecular effects in individual cells. Others use discrete models within individual agents: Gerlee and Anderson^40^ used small neural networks to simulate individual cell phenotypic "decisions" on the basis of microenvironmental inputs, whereas PhysiBoSS^41^ combines the Boolean network modeling approach of MaBoSS^42,43^ with PhysiCell^21^ to simulate molecular processes in individual cells.

## EXAMPLES OF CELL-BASED MODELING IN CANCER BIOLOGY

We now explore a series of modeling themes that illustrate the use of cell-based modeling in cancer biology. Although we cannot comprehensively review all cell-based modeling in cancer (or even sample all major use cases for cell-based modeling), these themes are drawn from across the field to demonstrate scientific problems with significant cell-scale effects where cell-based models can yield new insights.

### Hypoxia in Breast Cancer

**FIG 2.**
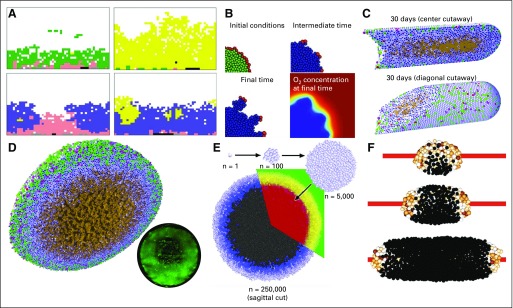
Cell-based models of hypoxia in breast cancer. (A) A cellular automaton model of breast cancer that explores cellular metabolic changes and early development of invasion. Reprinted with permission from Gatenby et al.^44^ (B) An immersed boundary model to simulate cancer invasion under hypoxic gradients. Adapted with permission from Anderson et al.^47^ (C) PhysiCell (a center-based model [CBM]) simulation of ductal carcinoma in situ as it advances in breast ducts under diffusive growth limits. Note the brown necrotic core. Adapted with permission from Ghaffarizadeh et al.^21^ (D) Adapted PhysiCell simulation of hanging-drop tumor spheroids. Oxygen diffusive limits lead to hypoxic gradients, greatest proliferation on the outer edge, an interior quiescent region, and an central necrotic core (brown). Note the network of fluid-filled pores that emerges from the necrotic core mechanics. These are observed in experiments. The inset shows a fluorescent image of a hanging-drop tumor spheroid. Adapted with permission from Ghaffarizadeh et al.^21^ (E) CBMs of tumor spheroids pioneered by Drasdo and Höhme produced similarly layered structures. Reprinted with permission from Drasdo and Höhme.^48^ (F) A CBM of tumor cords growing around a blood vessel and showing a reversed structure with viable tissue in the interior. Adapted with permission from Szymańska et al.^49^

Many groups have used cell-based models to investigate tumor growth in hypoxic tissues and more generally, the effect of diffusive transport limits. Gatenby et al^44^ and Smallbone et al^45^ used CAs to examine hypoxia-driven switching to invasive phenotypes in ductal carcinoma in situ (DCIS). They incorporated cellular metabolic adaptations to hypoxia, which allowed them to study early tumor invasion (Fig 2A). Anderson and colleagues^46,47^ extended earlier CA results by adapting IBCell^30^ (an IBM) to mouse mammary (EMT6/Ro) tumor cell proliferation in hypoxic tissues. As before, they found that hypoxic gradients could drive tissue invasion, but IBCell’s improved modeling of cell adhesion and biomechanics predicted more rounded invasive tips^47^ (Fig 2B).

Macklin et al^50^ and Hyun and Macklin^51^ applied a CBM to study oxygen-driven proliferation and necrosis in solid-type DCIS with comedonecrosis. After calibrating to individual patient pathology data (tissue specimens immunostained for the Ki67 protein to detect cycling cells, cleaved caspase 3 to detect apoptosis, and annotated with viable rim sizes and cell density^50^), they were able to simulate comedonecrosis and microcalcifications as emergent properties of the simulations along with realistic, constant rates of tumor advancement along the breast ducts. Ghaffarizadeh et al^21^ refined the DCIS model and extended it to 3D as well as simulated the hypoxic interiors of hanging drop spheroids calibrated to match MCF-10A birth and death kinetics in culture (Figs 2C and D). As in early 3D work by Drasdo and Höhme^48^ on EMT6/Ro cells (Fig 2E), they predicted a layered structure—an outer proliferative rim surrounding a quiescent perinecrotic region and an interior necrotic core. They were the first to predict networks of fluid-filled pores in the necrotic cores that emerge from the competing effects of necrotic cell shrinking and adhesion; these structures are observed in experimental models (Fig 2D inset). Szymańska et al^49^ used a CBM of EMT6 cells to simulate a growing tumor cord—a solid tumor that grows around a blood vessel. They predicted a similar three-layer structure but in reverse order—a proliferating core nearest the blood vessel, quiescent interior, and necrotic exterior (Fig 2F).

### Tumor-Induced Angiogenesis and Drug Delivery

**FIG 3.**
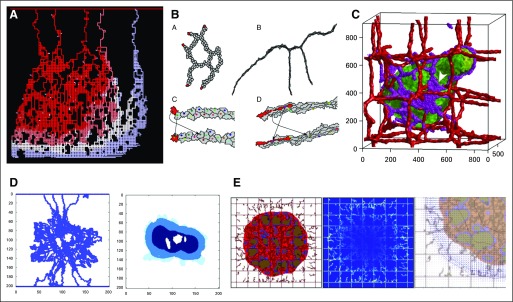
Tumor-associated angiogenesis and vascular flow. (A) A two-dimensional (2D) cellular automaton model of sprouting angiogenesis used to study drug delivery from tumor vasculatures. Reprinted with permission from McDougall et al.^53^ (B) A 2D cellular Potts model of angiogenesis. Stalk cells can overtake tip cells to become new tip cells. The arrows show these role swaps. Reprinted with permission from Boas and Merks.^55^ (C) A 3D cellular Potts model of sprouting angiogenesis driven by vascular endothelial growth factor released by hypoxic tumor cells. Adapted with permission from Shirinifard et al.^56^ (D) A 2D cellular automaton model (left) to investigate drug delivery to simulated tumors (right). Adapted with permission from Cai et al.^57^ (E) A discrete angiogenesis model of McDougall et al^53^ combined with a continuum tumor growth model^58^ used to investigate the effect of interstitial fluid pressure and lymphatic drainage on therapeutic delivery. Shown are tumor and the discrete vasculature (left); fluid extravasation from blood and lymphatic vessels (middle); and interstitial fluid velocity (right), which hinders drug delivery. Adapted with permission from Wu et al.^59^

Tumor-induced angiogenesis allows lesions to grow to clinically detectable sizes.^3^ McDougall and colleagues^52,53^ modeled sprouting angiogenesis with a CA model of vessel tip migration: Sprout tip agents followed chemotactic and haptotactic signals to migrate toward hypoxic tumor regions and left a trail of functional vessels. They incorporated a detailed vascular network flow model, including dynamic wall shear stress rules for vessel branching and anastomosis (vessel looping), and used this framework to explore therapeutic delivery from tumor-associated vasculatures (Fig 3A). Bauer et al^54^ used a CPM to simulate tumor-induced angiogenesis, by adding a detailed microenvironment, including extracellular matrix (ECM) and multiple vascular endothelial growth factor isoforms; they concluded that variations in the spatial distributions of proangiogenic factors greatly affect capillary morphology and that inhomogeneities in nonvascular tissue naturally lead to capillary anastomosis. Boas and Merks^55^ used a CPM to investigate novel hypotheses on cell overtaking: Cell-cell biomechanical and chemical communication can cause endothelial cells in the stalk to assume the role of migrating tip cells by migrating to the front of an advancing vessel (Fig 3B). Shirinifard et al^56^ used a CPM to investigate tumor growth with angiogenesis and showed that tumor size increases with increasing angiogenesis and that the tumors grow along the vasculature (Fig 3C).

Cai et al^57^ used a CA model of tumor cells in a continuous ECM coupled with a discrete angiogenesis model that included flow effects and substrate perfusion from the vasculature (Fig 3D). They showed that the final vessel configuration depends on emergent, dynamic feedback mechanisms in vascular remodeling rather than on initial conditions. Wu et al^59^ extended an earlier hybrid discrete-continuum model^58^ (that was based on that of McDougall and colleagues^52,53^) to investigate the influence of interstitial fluid pressure, interstitial fluid flow, and lymphatic drainage on drug delivery in growing tumors.^59^ They found that elevated interstitial hydraulic conductivity and high interstitial fluid pressure limit the transvascular delivery of nutrients and therapeutics (Fig 3E).

### Cancer Stem Cells

**FIG 4.**
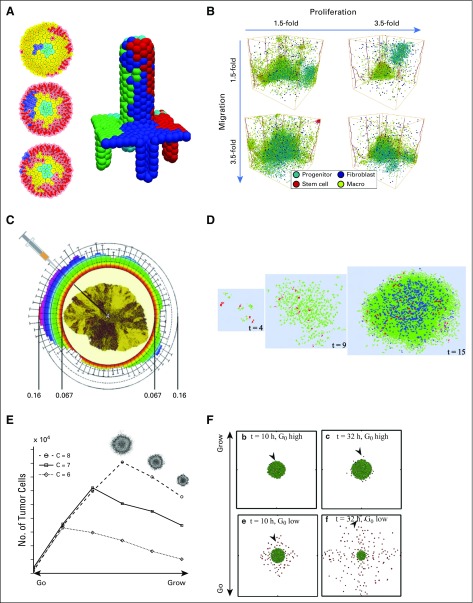
Cancer stem cells, invasion, and the "go or grow" hypothesis. (A) Top view of a three-dimensional (3D) center-based model of colon crypts (left plots) where the stem-cell niche is in the center. A nonstem mutation (blue cells) is swept out of the crypt by the proliferative cell flux. On the right, is a 3D view of four such ducts that feed cells to a central villus, which is based on the same simulation model. Adapted with permission from Fletcher et al^61^ (left) and Mirams et al^37^ (right). (B) A 3D cellular automaton (CA) model (with stem-cell effects) of how chemical signaling with fibroblasts and macrophages can drive triple-negative breast cancer. Among these findings, if stromal cells can promote increased cancer cell migration, the overall tumor grows. Reprinted with permission from Norton et al.^62^ (C) A 2D CA model to investigate the spread of traits in growing tumors, when cancer cells and their progeny could carry four tumor traits. Traits disseminate largely radially, with clear implications for tumor needle biopsies. Adapted from Poleszczuk and Enderling.^11^ (D) A 2D cellular Potts model of stem cells in glioblastoma that shows their role in building resistance to radiotherapy. Reprinted with permission from Gao et al.^63^ (E) Lattice gas CA models of the "go or grow" hypothesis in glioblastoma multiforme. As cells spend more time proliferating, they contribute to better growth up to a critical transition point; beyond this point, decreased migration is insufficient to open space for cell division. Adapted with permission from Hatzikirou et al.^64^ (F) A center-based model to explore the "go or grow" hypothesis in glioblastoma multiforme. Here, G_0_ is the models' growth rate parameter. Adapted with permission from Kim et al.^65^

Models of cancer stem cells (CSCs) offer valuable insights into the driving forces of cancer biology. Fletcher and colleagues^37,60,61^ developed a 3D CBM of colonic crypts to explore the role of stem cells (in the bottom of the crypt) in colorectal carcinogenesis. Neighboring cells were connected by linear springs, and stem-cell division and differentiation were driven by Wnt gradients along the crypt axis. The geometry of the stem-cell hierarchy (proliferation at the crypt base, expansion and differentiation along the middle and top) created an overall base-to-top proliferative cell flux. This flux has an anticancer protective effect wherein it pushes any mutated cell and its progeny out of a crypt before they can spread throughout a crypt, unless the mutation occurs in a stem-cell niche (Fig 4A).

Norton et al^62^ built a 3D CA model to examine the interaction between triple-negative breast cancer and stromal cells. Stem cells proliferated and differentiated into progenitor cells, and cancer cells exchanged chemical signals with fibroblasts and infiltrating macrophages. Among their results, they found that increasing the stromal effect on cancer cell proliferation decreased overall tumor size, whereas increasing the stromal effect on cancer cell migration increased tumor size (Fig 4B).

Poleszczuk et al^66^ developed a 2D CA model of CSCs and nonstem cancer cells, which tracked four traits in each individual cell: migration rate, apoptosis, symmetric CSC division, and cancer cell proliferation potential. They found that increasing the cancer cell proliferation potential could reduce tumor growth because the increased cancer cell population competed with CSCs for space and inhibited CSC division. They also found that traits propagated radially from the centers of growing tumors; this has implications for biopsies of tumor heterogeneity (Fig 4C). Gao et al^63^ used a CPM to investigate the role of glioma stem cells (GSCs) in glioblastoma growth and radiation therapy response (Fig 4D). They found that switching from asymmetric to symmetric division or fast GSC cycling was necessary to explain clinical observations of glioma repopulation after radiotherapy and that the expanded GSC fraction could reduce radiosensitivity.

Alfonso et al^67^ also explored radiotherapy treatment paradigms with respect to a heterogeneous population of CSCs and cancer cells using a 3D CA model. They found that CSCs, which are typically more radioresistant, segregated to the center of the tumor across a range of proliferation and death parameters. This emergent phenomenon is due to the faster cycling time of the cancer cells compared with CSCs. When these cell arrangements were subjected to radiotherapy, they found that radiotherapy is more effective at tumor control when it is concentrated on the tumor center where CSCs are located rather than when it is spread homogeneously across the entire tumor.

### The "Go or Grow" Hypothesis for Glioblastoma Multiforme

Tektonidis et al^68^ examined the "go or grow" (GoG) hypothesis in gliomas, where tumor cells must make a "decision" between migration (go) or proliferation (grow). They modeled data from 3D spheroid cell cultures with a 2D LCGA model and attempted to recapitulate three experimental observations: nonidentical spreading rates of the invasive rim and central core, radially persistent and symmetric cell motion, and a highly proliferative central core compared with the remaining tumor. Tektonidis et al evaluated the emergent model behavior under a variety of cell phenotype rule sets to determine which rules were required to predict the three observations. They found that a proliferative-motility dichotomy (the GoG hypothesis), cell-cell repulsion, and density-dependent switching between the proliferative and motile states were required to match experimental observations. They concluded that disruption of the GoG mechanism to favor proliferation could limit the required tumor resection volume in surgical interventions.

Hatzikirou et al^64^ investigated the GoG hypothesis in glioblastoma multiforme (GBM) using a 2D LGCA model. In their work, cells could divide, re-orient, migrate, or apoptose on the basis of local oxygenation conditions. They modeled the GoG hypothesis by switching hypoxic cells to a motile phenotype and reverting to a proliferative phenotype after escaping hypoxia. They found that increasing the cell bias toward proliferation increases overall tumor growth, but crossing a threshold could decrease overall tumor growth when motility is insufficient to open new space for cell division (Fig 4E). Comparable results were obtained by Gerlee and Nelander^69^ using stochastic switching between the two phenotypes (migrate or proliferate). From their CA model, they derived a system of coupled PDEs to investigate further the relationships between cell-level parameters and tumor-scale dynamics.

In related work, Böttger et al^70^ explored a 2D LGCA GoG model for GBM to provide a more quantitative parameter space analysis of a tumor’s invasive dynamics. Systematically varying model parameters for proliferation and motility led to counterintuitive results about invasion. Specifically, invasion speed depended on two competing processes: emptying space as a result of cell migration and filling space as a result of cell proliferation. In later work, Böttger et al^71^ used similar techniques to find that if cell motility decreases with increased cell density, then small tumors self-extinguish. On the other hand, increasing cell motility with increasing cell density leads to self-sustaining growth, similar to the Allee effect frequently observed in ecology.

Kim et al^65^ used a CBM to explore GBM using miR-451 as an intracellular detector of glucose, with an ODE model of miR-451 as the effector for selecting migration versus proliferation for glioma cells (Fig 4F). They found that cell migration depended not only on glucose, but also on mechanical spacing between cells. They also predicted that the placement of chemoattractants at the edges of a resected tumor could reduce GBM cell migration from the resection site. These GoG examples highlight the key role played in single-cell decisions in GBM and the potential for cell-based models that explore the clinical behaviors that emerge from single-cell effects.^72^

### Cancer Invasion and Epithelial-Mesenchymal Transition

Cancer invasion is essential to metastatic progression.^3,72-74^ Cancer cells acquire a motile phenotype to escape primary tumors and invade nearby tissues (as conceptually modeled in epithelial-to-mesenchymal transition [EMT]^75^), invade and travel within blood and lymphatic vessels,^76,77^ and finally colonize distant metastatic niches.^78^ Because single-cell effects are critical, many cell-based models have investigated cancer invasion with or without explicit modeling of EMT.

Reher et al^79^ developed a 2D LGCA model to simulate the effects of a heterogeneous cell-cell adhesion in an epithelial layer, with decreased cell-cell adhesion representing one of the effects of EMT. Cell-cell adhesion was modeled by varying the initial and maximum number of adhesion receptors for each virtual cell. They found that increased adhesion heterogeneity as well as decoupling receptor number from environmental signals (cell-cell contact) lead to increased dissemination.

**FIG 5.**
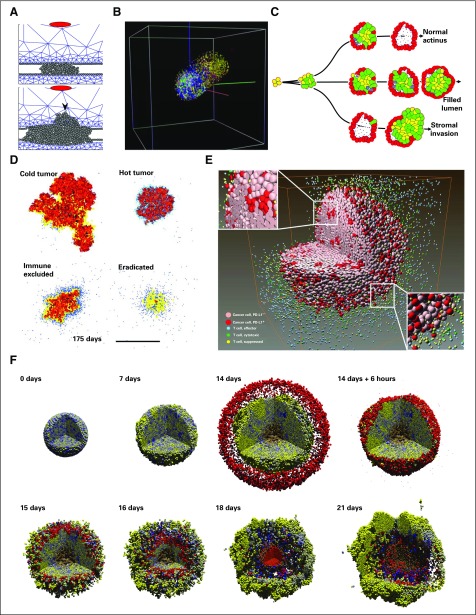
Cancer invasion and immunosurveillance. (A) In a center-based model, signals secreted by stromal cells (red) induce tumor cells (gray) to degrade the basement membrane and invade the stroma (blue mesh). Adapted with permission from Kim et al.^80^ (B) A three-dimensional (3D) cellular automaton (CA) model to study selection in heterogeneous brain cancers. Cells could mutate their signaling network parameters, which leads to more invasive clones. Adapted with permission from Zhang et al.^81^ (C) An immersed boundary method of contact-based signaling and polarization in breast acini.^82,83^ Cells with altered signaling could fill the lumen or invade the stroma. Adapted with permission from Anderson et al.^82^ (D) A 2D CA model of tumor-immune interactions. Immune cells (blue dots) become exhausted after too many successful tumor cell kills and create fibrotic tissue (yellow). Tumor encapsulation, tumor elimination, and chronic response are observed in the model. Adapted with permission from Kather et al.^84^ (E) A sophisticated 3D CA model of treatments targeting programmed cell death-1 (PD-1) and programmed death ligand 1 (PD-L1) in cancer cells. (PD-L1^+^ cells express PD-L1; PD-L1^−^ cells do not.) Reprinted with permission from Gong et al.^85^ (F) A 3D center-based model of immune responses to an immunostimulatory factor in a heterogeneous tumor (shaded by immunogenicity; yellow cells are most immunogenic). Immune cells (red) seek and adhere to cancer cells, test for immunogenicity, and induce apoptosis. The immune response failed after immune cells aggregated near a local maximum in the signaling factor, which allows the tumor to repopulate. Adapted with permission from Ghaffarizadeh et al.^21^ This work was explored further with high-performance computing.^10^

Kim and Othmer^80^ developed a center-based model with a continuous ECM description to investigate the interactions of tumor cells, signal-secreting stromal cells, and the ECM. Stromal levels of fibroblast-secreted protein initiated EMT, which switches cells to an invasive, motile phenotype. Invasive cells also secreted a tumor-associated protease, which degrades the basement membrane and ECM. Degradation of the basement membrane results in more cell exposure to fibroblast-secreted protein, which results in more phenotypic switching; more invasion; more ECM degradation; and ultimately, collective cell invasion (Fig 5A). These results suggest that inhibiting fibroblast secretions could affect invasive potential.

Zhang et al^81^ used a 3D CA model to investigate tissue invasion and tumor cell heterogeneity in glioma. Using a subcellular signaling network, cells divide and move on the basis of external concentrations of nutrients and signaling molecules. They acquire oncogenic mutations upon division to produce new clones. Each successive clone is more proliferative and nutrient seeking, which increases overall invasive potential. By starting with a sphere of the least oncogenic cells in the center of the simulation, Zhang et al found that heterogeneity increased in all regions of the simulation followed by a decrease in heterogeneity in the regions that contain the nutrient source and eventual recovery of heterogeneity. The decrease was attributed to an outgrowth of the most oncogenic subclone, which potentially explains the asymmetric invasive growth seen in experimental and clinical reports (Fig 5B).

Anderson et al^82^ used an IBM to investigate morphologic changes in breast acini as a result of variation in cell polarization and anoikis behaviors in response to cell contact with other cells and the basement membrane. In this, and additional work by Rejniak et al,^83^ Anderson et al showed that atypical behavior in a single cell can lead to eventual intraductal and stromal invasion (Fig 5C).

In a sophisticated investigation of metastatic colonization, Araujo et al^86^ developed a hybrid CA model of prostate cancer (PCa) bone metastasis. In their work, mesenchymal stromal cells differentiated into osteoblasts that created bone material, whereas osteoclasts degraded bone. In the absence of tumor cells, these cell populations coordinated through transforming growth factor-β and receptor activator of nuclear factor kappa beta ligand (RANKL) signaling to maintain healthy bone tissue. When PCa cell agents were introduced into the bone, tumor-secreted transforming growth factor-β promoted osteoblast activity, but osteoblast-osteoclast feedbacks simultaneously degraded bone to release new growth factors that could drive additional PCa proliferation and invasion. Araujo et al studied the model to compare potential improvements of RANKL inhibitors and bisphosphonates (two standard-of-care treatments for bone metastases). They found that additional refinements to bisphosphonates would yield little clinical improvement over current treatments, whereas improvement of RANKL inhibitors from the current (model-estimated) 40% inhibition closer to a theoretical maximum 100% inhibition could dramatically improve outcome.

### Tumor Immunosurveillance

Individual interactions between tumor cells and immune cells are critical to immunosurveillance.^3,7,87^ Cell-based models are uniquely capable of examining these while considering the roles of stochasticity and heterogeneity.

Kather et al^84^ developed a CA model to study complex interactions between tumor cells and immune cells. In their model, immune cells could randomly appear (an influx model), migrate toward tumor cells, and kill them. Immune cells were assumed to be exhausted after killing a maximum number of times and would induce tissue fibrosis that impaired cell migration. Tumor cells could proliferate, die, remain stationary, or migrate on the basis of the number of open neighboring lattice sites and their distance to the nearest open lattice site (a phenomenologic model of necrosis). Depending on the relative migration and proliferation parameters for tumor and immune cells, this model could exhibit diverse tumor-immune interactions, including successful immunosurveillance (eradicated), tumor encapsulation by fibrotic tissue (immune excluded), and ongoing immune responses (Fig 5D). Gong et al^85^ recently developed a 3D CA model to investigate the tumor-immune responses to programmed cell death-1 and programmed death-ligand 1 inhibition (Fig 5E).

Ghaffarizadeh et al^21^ developed a 3D off-lattice model of an immune attack on a tumor with a heterogeneous oncoprotein (mutations increased both proliferation and immunogenicity). After simulating initial growth, they added simulated immune cells that chemotaxed toward tumor-released immunostimulatory factors. Each immune cell tested for mechanical collision with cells, formed an (Hookean) adhesion, tested for immunogenicity, and stochastically attempted to induce tumor cell apoptosis. Attached immune cells eventually would succeed in killing the tumor cell and detach or continue the attempt before detaching and continuing their search for new targets. Their simulations showed initial tumor regression but eventual tumor regrowth when immune cells passed some tumor cells and formed large clumps near maxima of the immunostimulatory factor (Fig 5F). The authors have expanded their investigation to supercomputers to explore further the effect of stochastic migration on the overall efficacy of the immune response^10^; this work showcases the potential for using supercomputers to explore large therapeutic design spaces in high throughput.

## ADDITIONAL RESOURCES

As companions to this review, we maintain three online curated collections:

1. Cloud-hosted examples of cell-based cancer models: https://www.scienceopen.com/collection/online-cancer-simulators
2. Open source cell-based simulation frameworks: https://www.scienceopen.com/collection/open-source-agent-frameworks-biology
3. Additional reviews of mathematical modeling in cancer: https://www.scienceopen.com/collection/math-modeling-in-cancer-reviews

## DISCUSSION

Cell-based methods can track single-cell traits and individual behaviors, which make them well suited for problems where single-cell effects are important, such as stem-cell hierarchies, heterogeneity, invasion, and tumor-immune interactions. They are ideal for hypothesis-driven computational experiments because biologic observations of single-cell behavior can be directly translated to agent rules.

Lattice-based methods are straightforward to implement and fast, which makes them useful for quickly testing new ideas. We show how lattice-based methods have yielded insights on cancer metabolism, cancer stem cells, angiogenesis, the GoG hypothesis, invasion, and cancer immunosurveillance. CA methods remain the most common method for modeling vascular networks, whereas CPMs have investigated the finer details of angiogenesis.

Lattice-based methods are at risk for grid-based artifacts, and their biomechanical realism is limited; off-lattice models can readily incorporate biomechanics and off-lattice cell-cell interactions. As the hypoxia and immunosurveillance examples show, novel structures can arise from the interplay of stochasticity, transport limitations, mechanics, and single-cell characteristics. However, off-lattice models often are computationally demanding, and they generally have many parameters to calibrate. Ultimately, no single method is best for all problems. Modelers should reproduce findings with multiple approaches to avoid algorithm-dependent biases.^88^

This is an exciting time to apply cell-based modeling to cancer. Increases in computational power are allowing larger simulation studies with greater sophistication, and high-throughput computing is enabling exploration of high-dimensional parameter spaces.^10^ Open source platforms have lowered the barrier to entry for using sophisticated techniques (Table 1). In the future, we envision that cell-based modeling software will be increasingly user friendly, cloud hosted, and open to modular contributions from the community,^88^ which would potentiate a community-driven ecosystem of interoperable tools that together exceed the sum of their parts.^1^ We are excited to imagine the new insights that are on the horizon.

## References

[1] Macklin P Frieboes HB Sparks JL et al Progress towards computational 3-D multicellular systems biology Adv Exp Med Biol 936 225 246 2016 27739051 10.1007/978-3-319-42023-3_12 PMC6590068

[2] Macklin P Biological background Cristini V Lowengrub J Multiscale Modeling of Cancer: An Integrated Experimental and Mathematical Modeling Approach Cambridge, United Kingdom Cambridge University Press 2010 8 23

[3] Hanahan D Weinberg RA Hallmarks of cancer: The next generation Cell 144 646 674 2011 21376230 10.1016/j.cell.2011.02.013

[4] Maley CC Aktipis A Graham TA et al Classifying the evolutionary and ecological features of neoplasms Nat Rev Cancer 17 605 619 2017 28912577 10.1038/nrc.2017.69 PMC5811185

[5] Zhang D Tang DG Rycaj K Cancer stem cells: Regulation programs, immunological properties and immunotherapy Semin Cancer Biolepub ahead of print on May 9, 2018 10.1016/j.semcancer.2018.05.001 PMC7859848 29752993

[6] McKeown SR Defining normoxia, physoxia and hypoxia in tumours-implications for treatment response Br J Radiol 87 20130676 2014 24588669 10.1259/bjr.20130676 PMC4064601

[7] Qu X Tang Y Hua S Immunological approaches towards cancer and inflammation: A cross talk Front Immunol 9 563 2018 29662489 10.3389/fimmu.2018.00563 PMC5890100

[8] Kalluri R The biology and function of fibroblasts in cancer Nat Rev Cancer 16 582 598 2016 27550820 10.1038/nrc.2016.73

[9] Macklin P When seeing isn’t believing: How math can guide our interpretation of measurements and experiments Cell Syst 5 92 94 2017 28837815 10.1016/j.cels.2017.08.005

[10] Ozik J Collier N Wozniak J et al High-throughput cancer hypothesis testing with an integrated PhysiCell-EMEWS workflow BMC Bioinformatics 19:483, 2018 30577742 10.1186/s12859-018-2510-x PMC6302449

[11] Poleszczuk J Enderling H A high-performance cellular automaton model of tumor growth with dynamically growing domains Appl Math (Irvine) 5 144 152 2014 25346862 10.4236/am.2014.51017 PMC4208695

[12] Tang J Enderling H Becker-Weimann S et al Phenotypic transition maps of 3D breast acini obtained by imaging-guided agent-based modeling Integr Biol 3 408 421 2011 10.1039/c0ib00092b PMC4009383 21373705

[13] Van Liedekerke P Palm MM Jagiella N et al Simulating tissue mechanics with agent-based models: Concepts, perspectives and some novel results Comput Part Mech 2 401 444 2015

[14] Lowengrub JS Frieboes HB Jin F et al Nonlinear modelling of cancer: Bridging the gap between cells and tumours Nonlinearity 23 R1 R9 2010 20808719 10.1088/0951-7715/23/1/r01 PMC2929802

[15] Macklin P Edgerton ME Discrete cell modeling Cristini V Lowengrub JS Multiscale Modeling of Cancer: An Integrated Experimental and Mathematical Modeling Approach Cambridge, United Kingdom Cambridge University Press 2010 88 122

[16] Poleszczuk J Macklin P Enderling H Agent-based modeling of cancer stem cell driven solid tumor growth Meth Mol Biol 1516 335 346 2016 10.1007/7651_2016_346 PMC6587968 27044046

[17] Deutsch A Dormann S Cellular Automaton Modeling of Biological Pattern Formation: Characterization, Applications, and Analysised 2 New York, NY Birkhauser 2017

[18] Wolf-Gladrow DA Lattice-Gas Cellular Automata and Lattice Boltzmann Models: An Introduction New York, NY Springer 2004

[19] Graner F Glazier JA Simulation of biological cell sorting using a two-dimensional extended Potts model Phys Rev Lett 69 2013 2016 1992 10046374 10.1103/PhysRevLett.69.2013

[20] Voss-Böhme A Multi-scale modeling in morphogenesis: A critical analysis of the cellular Potts model PLoS One 7 e42852 2012 22984409 10.1371/journal.pone.0042852 PMC3439478

[21] Ghaffarizadeh A Heiland R Friedman SH et al PhysiCell: An open source physics-based cell simulator for 3-D multicellular systems PLOS Comput Biol 14 e1005991 2018 29474446 10.1371/journal.pcbi.1005991 PMC5841829

[22] Sütterlin T Tsingos E Bensaci J et al A 3D self-organizing multicellular epidermis model of barrier formation and hydration with realistic cell morphology based on EPISIM Sci Rep 7 43472 2017 28262741 10.1038/srep43472 PMC5338006

[23] Dallon JC Othmer HG How cellular movement determines the collective force generated by the *Dictyostelium discoideum* slug J Theor Biol 231 203 222 2004 15380385 10.1016/j.jtbi.2004.06.015 PMC6457452

[24] Milde F Tauriello G Haberkern H et al SEM++: A particle model of cellular growth, signaling and migration Comput Part Mech 1 211 227 2014

[25] Newman TJ Modeling multicellular systems using subcellular elements Math Biosci Eng 2 613 624 2005 20369943 10.3934/mbe.2005.2.613

[26] Sottoriva A Kang H Ma Z et al A Big Bang model of human colorectal tumor growth Nat Genet 47 209 216 2015 25665006 10.1038/ng.3214 PMC4575589

[27] Sun R Hu Z Sottoriva A et al Between-region genetic divergence reflects the mode and tempo of tumor evolution Nat Genet 49 1015 1024 2017 28581503 10.1038/ng.3891 PMC5643198

[28] Fletcher AG Osterfield M Baker RE et al Vertex models of epithelial morphogenesis Biophys J 106 2291 2304 2014 24896108 10.1016/j.bpj.2013.11.4498 PMC4052277

[29] Alt S Ganguly P Salbreux G Vertex models: From cell mechanics to tissue morphogenesis Philos Trans R Soc Lond B Biol Sci 372 20150520 2017 28348254 10.1098/rstb.2015.0520 PMC5379026

[30] Rejniak KA An immersed boundary framework for modelling the growth of individual cells: An application to the early tumour development J Theor Biol 247 186 204 2007 17416390 10.1016/j.jtbi.2007.02.019

[31] Venugopalan G Camarillo DB Webster KD et al Multicellular architecture of malignant breast epithelia influences mechanics PLoS One 9 e101955 2014 25111489 10.1371/journal.pone.0101955 PMC4128597

[32] Novak IL Slepchenko BM A conservative algorithm for parabolic problems in domains with moving boundaries J Comput Phys 270 203 213 2014 25067852 10.1016/j.jcp.2014.03.014 PMC4107334

[33] Nickaeen M Novak IL Pulford S et al A free-boundary model of a motile cell explains turning behavior PLOS Comput Biol 13 e1005862 2017 29136638 10.1371/journal.pcbi.1005862 PMC5705165

[34] Ghaffarizadeh A Friedman SH Macklin P BioFVM: An efficient, parallelized diffusive transport solver for 3-D biological simulations Bioinformatics 32 1256 1258 2016 26656933 10.1093/bioinformatics/btv730 PMC4824128

[35] Szabó A Merks RMH Blood vessel tortuosity selects against evolution of aggressive tumor cells in confined tissue environments: A modeling approach PLOS Comput Biol 13 e1005635 2017 28715420 10.1371/journal.pcbi.1005635 PMC5536454

[36] Powathil GG Munro AJ Chaplain MAJ et al Bystander effects and their implications for clinical radiation therapy: Insights from multiscale in silico experiments J Theor Biol 401 1 14 2016 27084360 10.1016/j.jtbi.2016.04.010

[37] Mirams GR Arthurs CJ Bernabeu MO et al Chaste: An open source C++ library for computational physiology and biology PLOS Comput Biol 9 e1002970 2013 23516352 10.1371/journal.pcbi.1002970 PMC3597547

[38] Swat MH Thomas GL Belmonte JM et al Multi-scale modeling of tissues using CompuCell3D Methods Cell Biol 110 325 366 2012 22482955 10.1016/B978-0-12-388403-9.00013-8 PMC3612985

[39] Sütterlin T Kolb C Dickhaus H et al Bridging the scales: Semantic integration of quantitative SBML in graphical multi-cellular models and simulations with EPISIM and COPASI Bioinformatics 29 223 229 2013 23162085 10.1093/bioinformatics/bts659

[40] Gerlee P Anderson AR Evolution of cell motility in an individual-based model of tumour growth J Theor Biol 259 67 83 2009 19285513 10.1016/j.jtbi.2009.03.005 PMC2706369

[41] Letort G Montagud A Stoll G et al 2018 https://academic.oup.com/bioinformatics/advance-article/doi/10.1093/bioinformatics/bty766/5087713

[42] Stoll G Viara E Barillot E et al Continuous time Boolean modeling for biological signaling: Application of Gillespie algorithm BMC Syst Biol 6 116 2012 22932419 10.1186/1752-0509-6-116 PMC3517402

[43] Stoll G Caron B Viara E et al MaBoSS 2.0: An environment for stochastic Boolean modeling Bioinformatics 33 2226 2228 2017 28881959 10.1093/bioinformatics/btx123

[44] Gatenby RA Smallbone K Maini PK et al Cellular adaptations to hypoxia and acidosis during somatic evolution of breast cancer Br J Cancer 97 646 653 2007 17687336 10.1038/sj.bjc.6603922 PMC2360372

[45] Smallbone K Gatenby RA Gillies RJ et al Metabolic changes during carcinogenesis: Potential impact on invasiveness J Theor Biol 244 703 713 2007 17055536 10.1016/j.jtbi.2006.09.010

[46] Anderson AR A hybrid mathematical model of solid tumour invasion: The importance of cell adhesion Math Med Biol 22 163 186 2005 15781426 10.1093/imammb/dqi005

[47] Anderson ARA Rejniak KA Gerlee P et al Microenvironment driven invasion: A multiscale multimodel investigation J Math Biol 58 579 624 2009 18839176 10.1007/s00285-008-0210-2 PMC5563464

[48] Drasdo D Höhme S A single-cell-based model of tumor growth in vitro: Monolayers and spheroids Phys Biol 2 133 147 2005 16224119 10.1088/1478-3975/2/3/001

[49] Szymańska Z Cytowski M Mitchell E et al Computational modelling of cancer development and growth: Modelling at multiple scales and multiscale modelling Bull Math Biol 80 1366 1403 2018 28634857 10.1007/s11538-017-0292-3

[50] Macklin P Edgerton ME Thompson AM et al Patient-calibrated agent-based modelling of ductal carcinoma in situ (DCIS): From microscopic measurements to macroscopic predictions of clinical progression J Theor Biol 301 122 140 2012 22342935 10.1016/j.jtbi.2012.02.002 PMC3322268

[51] Hyun AZ Macklin P Improved patient-specific calibration for agent-based cancer modeling J Theor Biol 317 422 424 2013 23084996 10.1016/j.jtbi.2012.10.017 PMC6607910

[52] McDougall SR Anderson AR Chaplain MA et al Mathematical modelling of flow through vascular networks: Implications for tumour-induced angiogenesis and chemotherapy strategies Bull Math Biol 64 673 702 2002 12216417 10.1006/bulm.2002.0293

[53] McDougall SR Anderson AR Chaplain MA Mathematical modelling of dynamic adaptive tumour-induced angiogenesis: Clinical implications and therapeutic targeting strategies J Theor Biol 241 564 589 2006 16487543 10.1016/j.jtbi.2005.12.022

[54] Bauer AL Jackson TL Jiang Y A cell-based model exhibiting branching and anastomosis during tumor-induced angiogenesis Biophys J 92 3105 3121 2007 17277180 10.1529/biophysj.106.101501 PMC1852370

[55] Boas SE Merks RM Tip cell overtaking occurs as a side effect of sprouting in computational models of angiogenesis BMC Syst Biol 9 86 2015 26589386 10.1186/s12918-015-0230-7 PMC4654812

[56] Shirinifard A Gens JS Zaitlen BL et al 3D multi-cell simulation of tumor growth and angiogenesis PLoS One 4 e7190 2009 19834621 10.1371/journal.pone.0007190 PMC2760204

[57] Cai Y Xu S Wu J et al Coupled modelling of tumour angiogenesis, tumour growth and blood perfusion J Theor Biol 279 90 101 2011 21392511 10.1016/j.jtbi.2011.02.017

[58] Macklin P McDougall S Anderson AR et al Multiscale modelling and nonlinear simulation of vascular tumour growth J Math Biol 58 765 798 2009 18781303 10.1007/s00285-008-0216-9 PMC3037282

[59] Wu M Frieboes HB McDougall SR et al The effect of interstitial pressure on tumor growth: Coupling with the blood and lymphatic vascular systems J Theor Biol 320 131 151 2013 23220211 10.1016/j.jtbi.2012.11.031 PMC3576147

[60] Fletcher AG Mirams GR Murray PJ et al Multiscale modeling of colonic crypts and early colorectal cancer Deisboeck TS Stamatakos GS Multiscale Cancer Modeling. CHAPMAN & HALL/CRC Mathematical and Computational Biology Series Boca Raton, FL CRC Press, Taylor & Francis Group 2010 111 134

[61] Fletcher AG Breward CJ Jonathan Chapman S Mathematical modeling of monoclonal conversion in the colonic crypt J Theor Biol 300 118 133 2012 22285553 10.1016/j.jtbi.2012.01.021

[62] Norton KA Jin K Popel AS Modeling triple-negative breast cancer heterogeneity: Effects of stromal macrophages, fibroblasts and tumor vasculature J Theor Biol 452 56 68 2018 29750999 10.1016/j.jtbi.2018.05.003 PMC6127870

[63] Gao X McDonald JT Hlatky L et al Acute and fractionated irradiation differentially modulate glioma stem cell division kinetics Cancer Res 73 1481 1490 2013 23269274 10.1158/0008-5472.CAN-12-3429 PMC3594421

[64] Hatzikirou H Basanta D Simon M et al ‘Go or grow’: The key to the emergence of invasion in tumour progression? Math Med Biol 29 49 65 2012 20610469 10.1093/imammb/dqq011

[65] Kim Y Kang H Lawler S The role of the miR-451-AMPK signaling pathway in regulation of cell migration and proliferation in glioblastoma Eladdadi A Kim P Mallet D Mathematical Models of Tumor-Immune System Dynamics New York, NY Springer 2014 125 156

[66] Poleszczuk J Hahnfeldt P Enderling H Evolution and phenotypic selection of cancer stem cells PLOS Comput Biol 11 e1004025 2015 25742563 10.1371/journal.pcbi.1004025 PMC4351192

[67] Alfonso JC Jagiella N Núñez L et al Estimating dose painting effects in radiotherapy: A mathematical model PLoS One 9 e89380 2014 24586734 10.1371/journal.pone.0089380 PMC3935877

[68] Tektonidis M Hatzikirou H Chauvière A et al Identification of intrinsic in vitro cellular mechanisms for glioma invasion J Theor Biol 287 131 147 2011 21816160 10.1016/j.jtbi.2011.07.012

[69] Gerlee P Nelander S The impact of phenotypic switching on glioblastoma growth and invasion PLOS Comput Biol 8 e1002556 2012 22719241 10.1371/journal.pcbi.1002556 PMC3375261

[70] Böttger K Hatzikirou H Chauviere A et al Investigation of the migration/proliferation dichotomy and its impact on avascular glioma invasion Math Model Nat Phenom 7 105 135 2012

[71] Böttger K Hatzikirou H Voss-Böhme A et al An emerging Allee effect is critical for tumor initiation and persistence PLOS Comput Biol 11 e1004366 2015 26335202 10.1371/journal.pcbi.1004366 PMC4559422

[72] Alfonso JCL Talkenberger K Seifert M et al The biology and mathematical modelling of glioma invasion: A review J R Soc Interface 14 14 2017 10.1098/rsif.2017.0490 PMC5721156 29118112

[73] Brabletz T Kalluri R Nieto MA et al EMT in cancer Nat Rev Cancer 18 128 134 2018 29326430 10.1038/nrc.2017.118

[74] Hanahan D Weinberg RA The hallmarks of cancer Cell 100 57 70 2000 10647931 10.1016/s0092-8674(00)81683-9

[75] Ye X Weinberg RA Epithelial-mesenchymal plasticity: A central regulator of cancer progression Trends Cell Biol 25 675 686 2015 26437589 10.1016/j.tcb.2015.07.012 PMC4628843

[76] Harney AS Arwert EN Entenberg D et al Real-time imaging reveals local, transient vascular permeability, and tumor cell intravasation stimulated by TIE2hi macrophage-derived VEGFA Cancer Discov 5 932 943 2015 26269515 10.1158/2159-8290.CD-15-0012 PMC4560669

[77] Juncker-Jensen A Deryugina EI Rimann I et al Tumor MMP-1 activates endothelial PAR1 to facilitate vascular intravasation and metastatic dissemination Cancer Res 73 4196 4211 2013 23687338 10.1158/0008-5472.CAN-12-4495 PMC3754905

[78] Aguado BA Bushnell GG Rao SS et al Engineering the pre-metastatic niche Nat Biomed Eng 1 1 2017 10.1038/s41551-017-0077 PMC5628747 28989814

[79] Reher D Klink B Deutsch A et al Cell adhesion heterogeneity reinforces tumour cell dissemination: Novel insights from a mathematical model Biol Direct 12 18 2017 28800767 10.1186/s13062-017-0188-z PMC5553611

[80] Kim Y Othmer HG A hybrid model of tumor-stromal interactions in breast cancer Bull Math Biol 75 1304 1350 2013 23292359 10.1007/s11538-012-9787-0 PMC6421845

[81] Zhang L Strouthos CG Wang Z et al Simulating brain tumor heterogeneity with a multiscale agent-based model: Linking molecular signatures, phenotypes and expansion rate Math Comput Model 49 307 319 2009 20047002 10.1016/j.mcm.2008.05.011 PMC2653254

[82] Anderson ARA Rejniak KA Gerlee P et al Modelling of cancer growth, evolution and invasion: Bridging scales and models Math Model Nat Phenom 2 1 29 2007

[83] Rejniak KA Wang SE Bryce NS et al Linking changes in epithelial morphogenesis to cancer mutations using computational modeling PLOS Comput Biol 6 6 2010 10.1371/journal.pcbi.1000900 PMC2928778 20865159

[84] Kather JN Poleszczuk J Suarez-Carmona M et al In silico modeling of immunotherapy and stroma-targeting therapies in human colorectal cancer Cancer Res 77 6442 6452 2017 28923860 10.1158/0008-5472.CAN-17-2006

[85] Gong C Milberg O Wang B et al A computational multiscale agent-based model for simulating spatio-temporal tumour immune response to PD1 and PDL1 inhibition J R Soc Interface 14 14 2017 10.1098/rsif.2017.0320 PMC5636269 28931635

[86] Araujo A Cook LM Lynch CC et al An integrated computational model of the bone microenvironment in bone-metastatic prostate cancer Cancer Res 74 2391 2401 2014 24788098 10.1158/0008-5472.CAN-13-2652 PMC4023121

[87] Yang Y Cancer immunotherapy: Harnessing the immune system to battle cancer J Clin Invest 125 3335 3337 2015 26325031 10.1172/JCI83871 PMC4588312

[88] Macklin P Key challenges facing data-driven multicellular systems biology 2018 https://arxiv.org/abs/1806.04736 10.1093/gigascience/giz127 PMC6812467 31648301

